# Modifiable risk factors for cancer in the middle East and North Africa: a scoping review

**DOI:** 10.1186/s12889-024-17787-5

**Published:** 2024-01-18

**Authors:** Razan Mansour, Abdallah Al-Ani, Maysa Al-Hussaini, Hikmat Abdel-Razeq, Akram Al-Ibraheem, Asem H. Mansour

**Affiliations:** 1 Department of Internal Medicine, University of Kansas Medical Center, Kansas, USA; 2 Department of Pathology and Laboratory Medicine, King Hussein Cancer Center, Amman, Jordan; 3 Department of Internal Medicine, King Hussein Cancer Center, Amman, Jordan; 4 Department of Nuclear Medicine, King Hussein Cancer Center, Amman, Jordan; 5 Department of Diagnostic Radiology, King Hussein Cancer Center, Amman, Jordan

**Keywords:** Risk factors, Middle East and North Africa, Tobacco, Alcohol, Physical inactivity, Diet, Environmental carcinogens, Infections, Prevention

## Abstract

**Purpose:**

This scoping review examines controllable predisposing factors attributable to cancer in the Middle East and North Africa (MENA) region’s adult population, highlighting opportunities to enhance cancer prevention programs.

**Design:**

We systematically searched the PubMed, Science Direct, and CINAHL, EMBASE, and Cochrane Library databases from 1997 to 2022 for articles reporting on the impact of modifiable risk factors on adult patients with cancer in the MENA region.

**Results:**

The review identified 42 relevant articles, revealing that tobacco consumption, obesity, physical inactivity, and diet are significant modifiable risk factors for cancer in the region. Tobacco smoking is a leading cause of lung, bladder, squamous cell carcinoma, and colorectal cancer. A shift towards a westernized, calorie-dense diet has been observed, with some evidence suggesting that a Mediterranean diet may be protective against cancer. Obesity is a known risk factor for cancer, particularly breast malignancy, but further research is needed to determine its impact in the MENA region. Physical inactivity has been linked to colorectal cancer, but more studies are required to establish this relationship conclusively. Alcohol consumption, infections, and exposure to environmental carcinogens are additional risk factors, although the literature on these topics is limited.

**Conclusion:**

The review emphasizes the need for further research and the development of targeted cancer prevention strategies in the MENA region.

**Supplementary Information:**

The online version contains supplementary material available at 10.1186/s12889-024-17787-5.

## Introduction

The Middle East and North Africa (MENA) region have experienced rapid growth in their economies, leading to social and cultural changes [1]. As a result, most economies in the Middle East are moving toward a westernized lifestyle associated with a diet rich in calories and limited physical activity. This shift is causing increased rates of obesity and lifestyle-related diseases, most important of which is cancer.

Cancer remains a global universal health problem attributable to reduced life expectancy [2]. In 2020, over 50% of the 20 million reported cancer cases worldwide resulted in death [3]. The burden of cancer is particularly relevant in the Middle East, where more than 430,000 new cancer cases were reported in 2020. Females in the Arab world are more affected by cancer than males, and recent evidence suggests that the elevated prevalence rates of female breast malignancy in the MENA region are due to increased rates of obesity and high consumption of a calorie-dense diet [3–6]. 

The literature shows that cancer is largely influenced by lifestyle and environmental factors [7]. In fact, the risk of cancer corresponds to the exposure to carcinogens over time. The major modifiable risk factors, such as smoking, alcohol, high BMI, insufficient physical activity, poor dietary intake, infections, and air pollution, were responsible for 164,780 cancer cases in adults above the age of 30 within the East Mediterranean region [8]. 

While the noted increase in cancer burden in the MENA region could be attributed to aging, improved diagnosis, and enhanced reporting of cases, the MENA region exhibited a significant increase in the prevalence of modifiable risk factors associated with cancer [9, 10]. Tobacco smoking is the most common substance abuse among adults in the Middle East, with recent studies indicating that 22% of young people aged 13–15 years engage in tobacco smoking [11]. Obesity significantly contributes to the global burden of cancer and disability-adjusted life years (DALY), with the MENA region experiencing a disproportionately high prevalence rate of obesity [4, 12]. Additionally, physical inactivity is prevalent in the MENA region, with recent studies indicating that about 47% of adults and 78% of adolescents do not engage in sufficient physical activity [13, 14]. 

The aforementioned risk factors were associated with significant increases in the burden of different cancers in the MENA region including but not limited to tracheal, lung, gastrointestinal, urinary, and oral cancers [8–10, 15–17]. This preventable burden poses a heavy economic strain on countries, especially those developing within the MENA region [18]. Moreover, such a preventable burden might also be hampered by the poor implementation of preventive interventions.

Therefore, this scoping review aims to map the existing evidence in the literature for controllable predisposing factors linked to cancer in the adult population of the MENA region, emphasizing the potential for enhancing cancer prevention programs. The study aims to (1) determine the relationship between cancer and intensity, type, amount, and frequency of tobacco and alcohol consumption; (2) find out if physical activity, diet, and obesity affect cancer in the MENA region; (3) explore the relationship between environmental or occupational exposure and cancer in the MENA region; *(4)* delineate gaps in the literature linking modifiable risk factors and cancer in the MENA region.

## Methods

### Evidence acquisition

This scoping review investigated the available studies on cancer risk factors, focusing on modifiable predisposing factors in the MENA region. The Arksey and O’Malley framework was used for the scoping review [19]. The research question was tested for eligibility using the PCC (Place, Concept, and Context) framework. In July 2022, a systematic literature search was conducted using the PubMed/MEDLINE, Science Direct, and CINAHL through EBSCOhost, EMBASE, and Cochrane/CENTRAL databases. Furthermore, the records of Google Scholar served as a supplementary resource to ensure a more comprehensive exploration of relevant studies. The search was manually conducted, and keywords were linked with Boolean operators. The results were then filtered by date and relevance. The current review focused on studies published before the year 2022 and after 1997, a period when the countries in the MENA region underwent significant economic, social, and cultural changes.

The search utilized the following keywords in isolation or combination: “Risk factors”, “Modifiable risk factors”, “Cancer”, “Adults, “Youth”, “Middle East”, “Gulf Council Countries”, and “Arab World Countries”. Moreover, the specific names of MENA region countries (e.g., Egypt, Iraq, Yemen, etc…) were incorporated into the search query. Moreover, relevant articles were screened through the bibliographies of relevant secondary researches including all previously published relevant narrative and scoping reviews. All processes, including systematic search conduction and documents, were conducted per the guidelines of the Preferred Reporting Items for Systematic Reviews and Meta-Analyses (PRIMSA) statement [20]. The detailed search strategy is illustrated in Supplemental Material A.

### Inclusion/exclusion eligibility criteria

Following the PICOS (Population, Intervention, Comparison, Outcome, and Study design) framework, the study’s eligibility criteria included studies reporting on modifiable risk factors linked to cancer among adults in the MENA region between 1997 and 2022. Given the scoping nature of the conducted review, qualitative, quantitative, and mixed study designs were included in the final evidence synthesis.

On the other hand, the study’s exclusion criteria involved studies that failed to fit the conceptual framework developed using the PCC model, articles published in any language other than English, studies conducted outside the MENA region, and studies that presented evidence for childhood cancers or had the study population exclusively below the age of 15 years. This decision aimed to differentiate the study from those concentrating on pediatric oncology, providing a clear delineation of the age groups under consideration. Moreover, certain study types, such as commentaries, conference abstracts, letters to the editors, or editorials, were excluded.

### Screening and data extraction

Two authors independently conducted an active search of databases using the eligibility criteria, cross-matching results to identify any discrepancies. All articles were underwent a primary screening process (i.e., by title and abstract) and a secondary screening process (i.e., by full-text content), and any discrepancies within the screening processes were handled by the senior author. The following data was extracted for all eligible articles: (1) study identifier, (2) country of origin, (3) characteristics of study population, (4) study design, (5) outcome measure(s) for predisposing factors attributable to cancer, (6) other relevant results to our study question, and (7) study limitations.

Additionally, data on the following predisposing factors were included: smoking and alcohol consumption, physical activity, diet, obesity, and environmental/occupational exposure. Data on environmental exposure included exposure to pollutants, toxins, or radiation in residential or community settings, living in proximity to industrial areas, waste disposal sites, or other locations where carcinogenic agents are present. Data on occupational exposure included professions that involve contact with substances known or suspected to be associated with an increased risk of cancer, including industrial workers, miners, and those working in carpeting, manufacturing or chemical processing plants.

Due to their relevance, tobacco and alcohol risk factors were examined in terms of intensity, type, and frequency; all of which are defined as follows: (1) intensity of tobacco consumption refers to the strength or concentration of tobacco smoke inhalation, which is measured either in terms of number of daily smoked cigarettes, nicotine content of consumed tobacco products, or smoking pack years. (2) intensity of alcohol consumption signifies the level of alcoholic beverage strength, which is measured in either self-reported drinking habits or standard drinking units. (3) Type of tobacco consumption included cigarettes, cigars, waterpipes (e.g., shisha), or smokeless tobacco products. (4) Type of alcohol consumption included but not limited to beer, wine, and spirits. (5) Frequency of tobacco or alcohol consumption is defined as how often does individuals consume the aforementioned (i.e., daily, weekly, monthly, annually, etc…).

### Quality assessment

The JBI critical appraisal tools for cross-sectional, case-control, cohort, and review studies were utilized to assess the quality of included evidence. The aforementioned tools are comprised of 8, 10, 11, and 11 items, respectively.

## Results

### Included papers

**Fig. 1:**
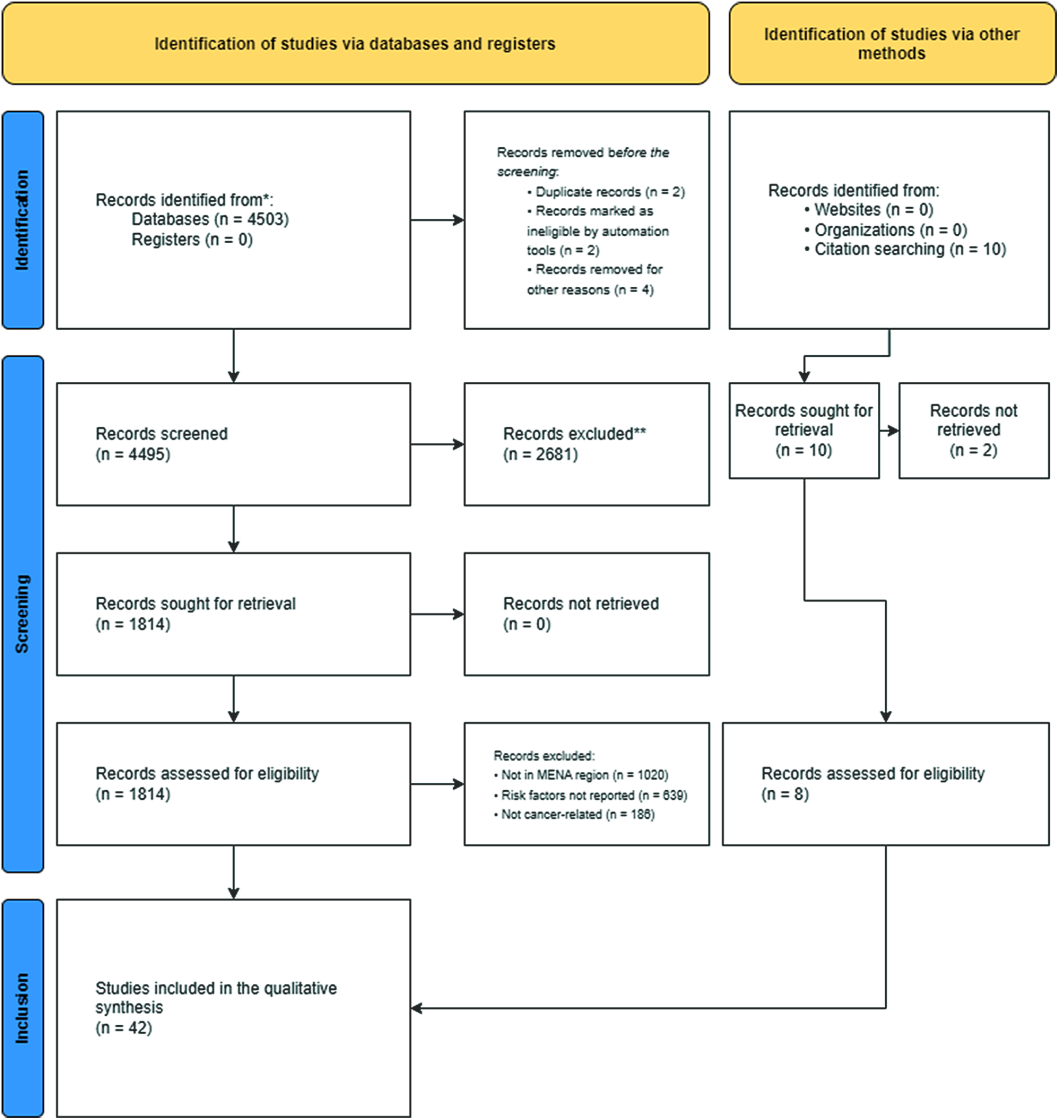
PRIMSA flowchart of included articles

**Fig. 2:**
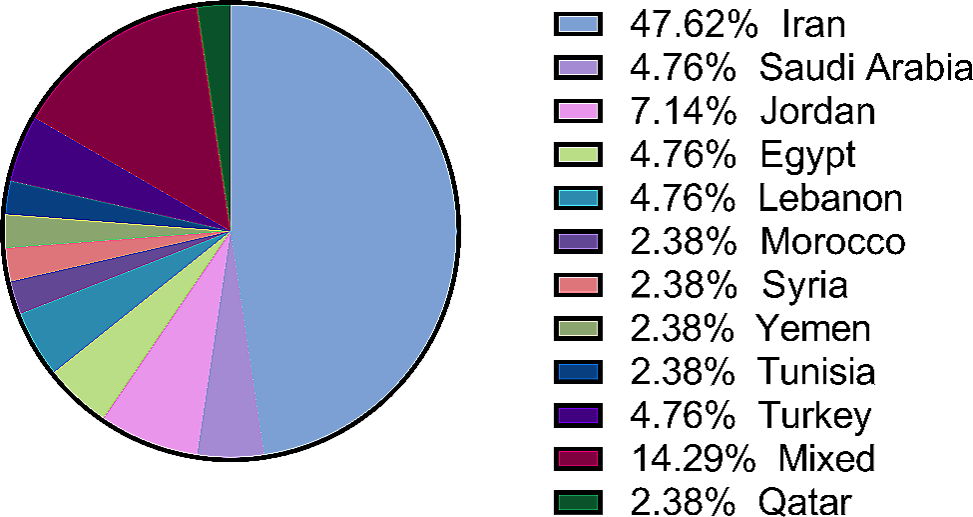
Geographical distribution of included studies

**Fig. 3:**
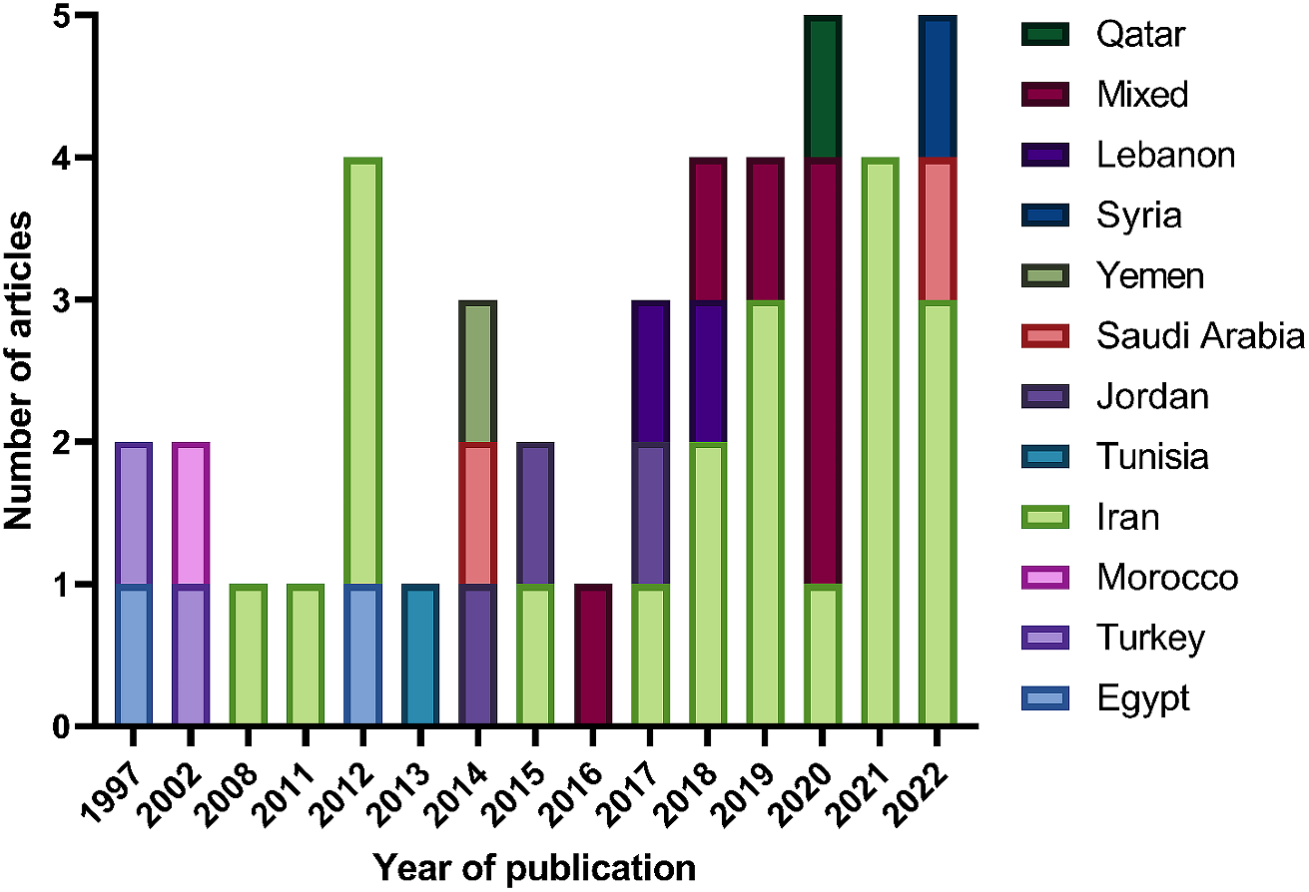
Number of publications regarding modifiable risk factors among cancer patients within the MENA region

**Fig. 4:**
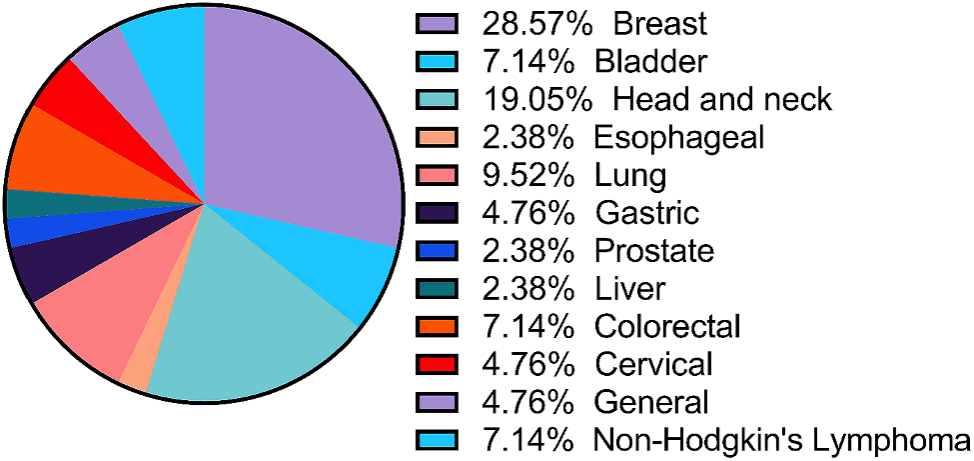
Distribution of studied cancer types

A total of 4495 records were included in the initial screening, and 184 were assessed for eligibility. The search and screening methods in this study identified 42 relevant articles for data extraction (Refer to Fig. 1). Tables 1 and 2 demonstrate the characteristics of included articles. The majority of these studies were from Iran (*n* = 20), [6, 21–39 followed by Jordan (*n* = 3), [40–42] with Egypt, Saudi Arabia, Turkey, and Lebanon each having two studies [43–50]. Morocco, Syria, Yemen, Tunisia, and Qatar each had one study [51–55]. Figure 2 showcases the geographical distribution of included nations. Included articles were published between 1999 and 2022, with the highest frequency of publications was recorded in 2022 (Refer to Fig. 3). Breast cancer was the most studied cancer (28.6%), followed by head and neck cancers (19.1%) (Refer to Fig. 4).

**Table 1:** Characteristics of included studies

Study Identifier	Study Design	Sample Size	Population Characteristics	Location & Time Period	Exposure/Independent Variable Tested	Outcome Measures
1. Azzeh et al. (2022)	Case-control	• *N* = 423 • Cases = 214 • Controls = 218	Post-menopausal Arab women with breast cancer	• Saudi Arabia • 2014–2016	• Dietary consumption of Mediterranean diet • BMI • Smoking status • Caloric intake • Prevalence of cancer	Odds ratio
2. Bedwani et al. (1997)	Case-control	• *N* = 308 • Cases = 151 • Controls = 157	Males with bladder cancer	• Egypt • 1994–1996	• Smoking status & characteristics	Odds ratio
3. Khlifi et al. (2013)	Case-control	• *N* = 520 • Cases = 169 • Controls = 351	Patients with head and neck cancers	• Tunisia • 2007–2008	• Occupational exposure (e.g., Chromium and Nickel)	Odds ratio
4. Nasrollahzadeh et al. (2008)	Case-control	• *N* = 871 • Cases = 300 • Controls = 571	Patients with esophageal squamous cell carcinoma	• Iran • 2003–2007	• Smoking status & characteristics • Other substances intake (e.g., Alcohol)	Odds ratio
5. Sasco et al. (2002)	Case-control	• *N* = 353 • Cases = 118 • Controls = 235	Patients with lung cancer	• Morocco • 1996–1997	• Smoking status & characteristics • Occupational exposure (e.g., asbestos, iron, nickel, silica, etc…) • History of respiratory disease	Odds ratio
6. Salarabadi et al. (2015)	Case-control	• *N* = 152 • Cases = 47 • Controls = 105	Patients with breast cancer	• Iran • 2012–2014	• BMI • Occupation • Physical activity • Consumption of calcium and vitamin D • Exposure to sunlight	Odds ratio
7. Hosseini et al. (2022)	Case-control	• *N* = 300 • Cases = 150 • Controls = 150	Patients with breast cancer	• Iran • 2017–2018	• Dietary intake • BMI • Physical activity • Smoking status • Other substances intake (e.g., Alcohol) • Food quality score (FQS)	Odds ratio
8. Narmcheshm et al. (2022)	Case-control	• *N* = 349 • Cases = 178 • Controls = 271	Patients with gastric cancer	• Iran • 2010–2012	• Smoking status & characteristics • BMI • Infections (e.g., H.Pylori) • Dietary intake	Odds ratio
9. Zheng et al. (2012)	Case-control	• *N* = 4602 • Cases = 1886 • Controls = 2716	Patients with bladder cancer	• Egypt • 2006–2010	• Smoking status & characteristics • Infections (e.g., H.Pylori, Schistosomiasis)	Odds ratio
10. Elkum et al. (2014)	Case-control	• *N* = 1172 • Cases = 534 • Controls = 638	Patients with breast cancer	• Saudi Arabia • 2007–2012	• Dietary intake • BMI • Smoking status & characteristics • Other substances intake (e.g., Alcohol) • Family history of cancer	Odds ratio
11. Marzbani et al. (2019)	Case-control	• *N* = 620 • Cases = 212 • Controls = 408	Females with breast cancer	• Iran • 2013–2015	• Dietary intake	Odds ratio
12. Nasher et al. (2014)	Case-control	• *N* = 180 • Cases = 60 • Controls = 120	Patients with oral squamous cell carcinoma	• Yemen • 2009–2011	• Smoking status of “Shamma” • Other substances intake (e.g., Alcohol) • Infections (e.g., Herpes)	Odds ratio
13. Abdolahinia et al. (2021)	Case-control	• *N* = 300 • Cases = 100 • Controls = 200	Patients with bladder cancer	• Iran • 2020	• Smoking status & characteristics • Other substances intake (e.g., Alcohol, Opium) • Dietary intake • Occupational exposure • Family history of cancer	Odds ratio
14. Toorang et al. (2021)	Case-control	• *N* = 224 • Cases = 207 • Controls = 217	Patients with stomach cancer	• Iran • 2010–2012	• BMI • Dietary intake	Odds ratio
15. Fararouei et al. (2019)	Case-control	• *N* = 1010 • Cases = 505 • Controls = 505	Female patients with breast cancer	• Iran • 2014–2016	• BMI • Dietary intake • Physical activity • Smoking status • OCP usage	Odds ratio
16. Hajjar et al. (2022)	Case-control	• *N* = 303 • Cases = 103 • Controls = 200	Patients with breast cancer	• Iran • NA	• Dietary intake • Recommended Food Score (RFS)	Odds ratio
17. Habibi et al. (2019)	Case-control	• *N* = 82 • Cases = 41 • Controls = 41	Patients with viral hepatitis-linked hepatocellular carcinoma (HCC)	• Iran • 2015	• Aflatoxin B1 levels	Means
18. Ghiasvand et al. (2012)	Case-control	• *N* = 986 • Cases = 493 • Controls = 493	Post-menopausal women with breast cancer	• Iran • NA	• BMI • OCP usage • Duration of breastfeeding • Prevalence of cancer	Odds ratio
19. Dosemeci et al. (1997)	Case-control	• *N* = 3207 • Cases = 2378 • Controls = 829	Male patients with laryngeal or lung cancer	• Turkey • 1979–1984	• Smoking status & characteristics • Other substances intake (e.g., Alcohol)	Odds ratio
20. Shivappa et al. (2017)	Case-control	• *N* = 353 • Cases = 153 • Controls = 202	Patients with colorectal cancer	• Jordan • 2010–2012	• Dietary intake (e.g., Dietary Inflammation Index (DII))	Odds ratio
21. Simonian et al. (2018)	Case-control	• *N* = 437 • Cases = 187 • Controls = 250	Patients with colorectal cancer	• Iran • 2014–2015	• BMI • Smoking status & characteristics • Occupational history • Physical activity • NSAID usage	Odds ratio
22. Rafeemanesh Noet al. (2018)	Case-control	• *N* = 216 • Cases = 104 • Controls = 112	Patients with breast cancer	• Iran • 2010–2014	• Physical activity • Occupational history • Smoking status & characteristics	Odds ratio
23. Ghoreishy et al. (2021)	Case-control	• *N* = 1050 • Cases = 350 • Controls = 700	Female patients with breast cancer	• Iran • 2013–2015	• Dietary intake	Odds ratio
24. Kaya et al. (2002)	Case-control	• *N* = 140 • Cases = 70 • Controls = 70	Patients with non-Hodgkin lymphoma	• Turkey • NA	• Infections (i.e., hepatitis)	Odds ratio
25. Mazdak et al. (2012)	Case-control	• *N* = 190 • Cases = 95 • Controls = 95	Males with prostate cancer	• Iran • 2005–2009	• Smoking status • Other substances intake (e.g., Alcohol) • Dietary intake • Sexual behavior • Medications history	Odds ratio
26. Rezaeian et al. (2012)	Case-control	• *N* = 260 • Cases = 140 • Controls = 120	Patients with non-Hodgkin lymphoma	• Iran • 2007–2011	• Infections (i.e., hepatitis)	Odds ratio
27. Khodavandi et al. (2011)	Case-control	• *N* = 170 • Cases = 70 • Controls = 100	Patients with non-Hodgkin lymphoma	• Iran • NA	• Infections (i.e., hepatitis)	Odds ratio
28. Quadri et al. (2019)	Systematic Review & Meta-Analysis	• Cross-sectional studies (*n* = 3; pn = 1443) • Case-control (*n* = 3; pn = 534)	Patients with oral cancer	• Saudi Arabia, Yemen • 1984–2018	• Smoking status of “Shamma”	Odds ratio
29. Taha & Eltom (2018)	Literature review	**NA**	Patients with breast cancer	**NA**	**NA**	**NA**
30. Tanner & Cheung (2020)	Systematic review	• Case-controls (*n* = 8; pn = 4939) • None case-controls (*n* = 5; pn = 2198)	Patients with breast cancer	• Gulf Cooperation Council countries • 2004–2018	• BMI • Physical activity	Odds ratio
31. Al-Jaber et al. (2016)	Literature review	*N* = 9; pn = 3130	Patients with oral cancer	• Egypt, Iraq, Jordan, Libya, Saudi Arabia, Sudan, Yemen • 1985–2014	• Smoking status • Dietary intake	Pooled percentages
32. Alqahtani et al. (2020)	Systematic review	• Cohort (*n* = 6; pn = 12,964) • Cross-sectional (*n* = 2; pn = 9180) • Case-control (*n* = 2; pn = 647)	Patients with oropharyngeal cancer	• Gulf Cooperation Council countries • 1994–2014	• Smoking status & characteristics	Pooled percentages
33. Obeid et al. (2020)	Systematic Review & Meta-Analysis	• Set A (*n* = 39; pn = 6104) • Set B (*n* = 24; pn = 5114) • Set C (*n* = 100; pn = 1,272,362)	• A (studies on cervical cancer and HPV prevalence) • B (studies on HPV prevalence and abnormal cervical cytology) • C (HPV prevalence in the general population)	• MENA region • 1999–2019	• Infections (i.e., HPV)	Pooled prevalence
34. Etemadi et al. (2017)	Cohort (Prospective)	*N* = 50,045	Adult participants of the Golestan Cohort Study	• Iran • 2004–2008	• Smoking status & characteristics • Other substances intake (e.g., Alcohol)	Odds ratio
35. Charafeddine et al. (2017)	Cohort (Retrospective)	**NA**	Patients with cancer	• Lebanon • 2008–2018	• Smoking status • BMI • Dietary intake • Infections (e.g., H.Pylori) • Other substances intake (e.g., Alcohol)	Estimated population attribution fraction (e-PAF)
36. Dar et al. (2015)	Cohort (Retrospective)	*N* = 102	Patients with oral cancer	• Jordan • 1996–2008	• Smoking status of “Narghile”	Odds ratio
37. Wang et al. (2021)	Cohort (Prospective)	*N* = 48,421	Adult participants of the Golestan Cohort Study	• Iran	• Alternative Health Eating Index (AHIE) • Health Eating Index (HEI) • Dietary Approach to Stop Hypertension (DASH) • Alternative Mediterranean Diet (AMED)	Hazard ratio
38. Harfouch et al. (2022)	Cohort (Retrospective)	*N* = 1117	Patients with colorectal cancer	• Syria • 2014–2018	• Physical activity • Occupational history	Raw percentage
39. Mhawej et al. (2018)	Cohort (Retrospective)	*N* = 30	Patients with oropharyngeal squamous cell carcinoma	• Lebanon • 2010–2016	• Infections (e.g., HPV) • Smoking status • Other substances intake (e.g., Alcohol)	Raw percentage
40. Abdel-Salam et al. (2020)	Cohort (Retrospective)	*N* = 229	Patients with lung cancer	• Qatar • 2010–2014	• Smoking status	Hazard ratio
41. Al-Amad et al. (2014)	Cross-sectional	*N* = 102	Patients with oral cancer	• Jordan • 1996–2008	• Smoking status of “Narghile”	Odds ratio
42. Khodakarami et al. (2012)	Cross-sectional	*N* = 1651 Cases = 860 Controls = 791	Participants from the general population and patients with invasive cervical carcinoma	• Iran • 2002–2008	• Infections (e.g., HPV)	Odds ratio

**Table 2:** Results and limitations of included studies

Study Identifier	Results	Limitations	Quality score
1. Azzeh et al. (2022)	• Consuming 1–2 or 3–5 servings of dairy products were protective against breast cancer (OR = 0.178; 95%CI = 0.037–0.859 & OR = 0.038; 95%CI = 0.004–0.372, respectively). • Weekly consumption of 1–2 servings legumes were protective against breast cancer (OR = 0.043; 95%CI = 0.010–0.191). • Consuming 3–5 servings of fruits and vegetables were protective against breast cancer (OR = 0.161; 95%CI = 0.043–0.605). • Consuming 1–2 or 3–5 servings of fish and seafood were protective against breast cancer (OR = 0.211; 95%CI = 0.82–0.545 & OR = 0.072; 95%CI = 0.202–0.265, respectively). • Daily consumption of 1–2 or 3–5 cups of tea were protective against breast cancer (OR = 0.06; 95%CI = 0.010–0.371 & OR = 0.06; 95%CI = 0.009–0.395, respectively). • Daily consumption of 1–2 or more than 5 cups of coffee were protective against breast cancer (OR = 0.159; 95%CI = 0.031–0.812 & OR = 0.144; 95%CI = 0.028–0.736, respectively).	• Questionnaire-related biases (e.g., recall bias) • Limited sample size • Inclusion of postmenopausal women	8/10
2. Bedwani et al. (1997)	• Smoking was associated with a higher likelihood of bladder cancer (OR = 6.6; 95%CI = 3.1–13.9). • Higher duration of smoking was associated with higher risk of bladder cancer (e.g., + 40 years) (OR = 16.5; 95%CI = 6.1–45.2). • Smokers with a history of schistosomiasis were associated with developing bladder cancer (OR = 9.4; 95%CI = 2.9–30.0).	• Type of bladder cancer was not accounted for • Inclusion of only a hospital-based population • Recall bias	6/10
3. Khlifi et al. (2013)	• High blood Chromium was associated with higher risk of developing head and neck cancers (OR = 2.1; 95%CI = 1.3–3.5). • High blood Nickel was associated with higher risk of developing head and neck cancers (OR = 8.87; 95%CI = 5.2–15.2). • Smokers with or without occupational exposure were associated with head and neck cancers (OR = 25.1; 95%CI = 11.8–53.1 & OR = 6.2; 95%CI = 2.9–13.4, respectively).	• Limited sample size	7/10
4. Nasrollahzadeh et al. (2008)	• Using tobacco without concomitant opium was associated with esophageal squamous cell carcinoma (OR = 1.7; 95%CI = 1.1–2.7). • When stratified by drug type, “Nass” was associated with esophageal squamous cell carcinoma (OR = 2.9; 95%CI = 1.5–5.8). • Using more than one type of tobacco products was associated with esophageal squamous cell carcinoma (OR = 1.2; 95%CI = 1.2–3.9).	• Recall bias • Sampling from a single location	7/10
5. Sasco et al. (2002)	• Compared to non-smokers, current light and heavy smokers were associated with higher likelihood of lung cancer (OR = 18.5; 95%CI = 4.1–83.5 & OR = 26.1; 95%CI = 6.6–103.3, respectively). • Users of Hashish/kiff and snuff had a higher likelihood of lung cancer compared to none-users (OR = 6.7; 95%CI = 1.65–29.9). • Passive smoking, occupational exposure, cooking, or kitchen ventilation were not associated with lung cancer.	• Recall bias • Inclusion of only a hospital-based population	6/10
6. Salarabadi et al. (2015)	• Increased body coverage against sunlight (OR = 2.9; 95%CI = 1.2–6.8) and over 5 years of OCP consumption (OR = 3.1; 95%CI = 1.3–7.3) were associated with higher risk of breast cancer among postmenopausal women. • Consumption of solid animal oils (OR = 2.7; 95%CI = 1.4–5.3) and butter or margarine (OR = 1.7; 95%CI = 1.1–2.8) were associated with higher risk of breast cancer. • Reduced consumption of milk (OR = 2.2; 95%CI = 1.1–4.4), yogurt (OR = 4.3; 95%CI = 1.5–11.9), and calcium (OR = 4.9; 95%CI = 1.1–22.3) were associated with higher risk of breast cancer. • Lack of consumption of soya beans (OR = 5.4; 95%CI = 2.5–11.5), green vegetables (OR = 4.1; 95%CI = 1.3–13.3), cabbage (OR = 3.6; 95%CI = 15.4–8.6), carrots (OR = 7.5; 95%CI = 2.9–19.1), and peas (OR = 5.4; 95%CI = 2.5–11.7) were all associated with higher risk of breast cancer. • Weekly consumption of white meat was associated with higher risk of breast cancer (OR = 9.7; 95%CI = 1.1–89.1).	• Recall bias • Limited sample size • Questionable statistics and methodology	3/10
7. Hosseini et al. (2022)	• Participants with high FQS (≥ 28) were less likely to develop breast cancer (OR = 0.58; 95%CI = 0.34–0.97). • When stratified by menopause status, the association persisted for premenopausal women (OR = 0.45; 95%CI = 0.23–0.88) but not their postmenopausal counterparts (OR = 0.76; 95%CI = 0.30–1.93).	• Questionnaire-related biases (e.g., recall bias) • Lack of control for major confounders such as diet	6/10
8. Narmcheshm et al. (2022)	• A diet pattern consisting of pantothenic acid, riboflavin, zinc, animal protein, calcium, biotin, animal fat, vitamin b12, and cholesterol was associated with gastric cancer in men only (OR = 1.86; 95%CI = 1.03–3.34). • A diet pattern consisting of selenium, thiamin, carbohydrates, vegetable protein, vitamin E, vegetable fat, niacin, sodium, and total iron was associated with gastric cancer in men only (OR = 2.15; 95%CI = 1.13–4.09).	• Lack of controls over the age of 70 • Limited sample size	6/10
9. Zheng et al. (2012)	• Among men, history of schistosomiasis is associated with urothelial cell carcinoma (OR = 1.4; 95%CI = 1.2–1.7) and squamous cell carcinoma (OR = 1.4; 95%CI = 1.1–1.7). • Among women, history of schistosomiasis is associated with urothelial cell carcinoma (OR = 1.9; 95%CI = 1.2–2.9) and squamous cell carcinoma (OR = 1.9; 95%CI = 1.2–3.0). • Among men, waterpipe smokers (OR = 1.3; 95%CI = 1.0–1.8), cigarette smokers (OR = 1.8; 95%CI = 1.4–2.2), or smokers of both (OR = 2.9; 95%CI = 2.1–3.9) were associated with urothelial cell carcinoma. However, only dual users were associated with squamous cell carcinoma (OR = 1.8; 95%CI = 1.2–2.6). • For nontobacco men users, environmental tobacco exposure was associated with only urothelial cell carcinoma (OR = 2.5; 95%CI = 1.2–5.1).	• Lack of enough women for statistical analysis • Self-report data for schistosomiasis diagnosis • Recall bias	6/10
10. Elkum et al. (2014)	• Obesity (OR = 2.29; 95%CI = 1.68–3.13), family history of breast cancer (OR = 2.31; 95%CI = 1.60–3.32), usage of HRT (OR = 2.25; 95%CI = 1.65–3.08), and postmenopausal status (OR = 1.72; 95%CI = 1.25–2.38) were associated with higher likelihood of breast cancer. • Breastfeeding (OR = 0.53; 95%CI = 0.34–0.84) and higher educational levels (OR = 0.11; 95%CI = 0.07–0.17) were protective against breast cancer.	• Sampling from a single hospital • Sampling from a hospital-based population • Limited sample size	5/10
11. Marzbani et al. (2019)	• Compared to daily consumption, 2–3 serving per month of vegetables is associated with breast cancer (OR = 2.8; 95%CI = 1.7–4.5). • Consuming soft drinks (OR = 2.8; 95%CI = 1.9–4.3), industrially produced juices (OR = 2.7; 95%CI = 1.1–6.5), fats and oils (OR = 1.9; 95%CI = 1.3–3.0), fried foods (OR = 4.5; 95%CI = 2.1–9.4), and sweets (OR = 2.6; 95%CI = 1.7–3.9) were associated with breast cancer.	• Incomplete assessment of confounders • Questionnaire-related biases (e.g., recall bias)	5/10
12. Nasher et al. (2014)	• Ex- and current “Shammah” use were associated with higher likelihood of OSCC (OR = 12.6; 95%CI = 3.3–48.2 & OR = 39; 95%CI = 14.0–105.0, respectively). • The isolated usage of “Shammah” (OR = 149.5; 95%CI = 12.3–1812.0), in conjunction with “qat” (OR = 43.1; 95%CI = 7.0–266.0), or in conjunction with smoking (OR = 14.2; 95%CI = 2.9–69.0) were all associated with OSCC. • EBV, HPV-18, and HPV-16 were not associated with OSCC.	• Sampling from a single hospital • Sampling from a hospital-based population • Limited sample size • Questionnaire-related biases (e.g., recall bias)	6/10
13. Abdolahinia et al. (2021)	• Light and heavy cigarette smoking were associated with risk of bladder cancer (OR = 3.4; 95%CI = 1.3–8.9 & OR = 15.8; 95%CI = 5.9–42.4, respectively). • Light and heavy consumption of opium were associated with risk of bladder cancer (OR = 6.0; 95%CI = 2.3–15.5 & OR = 11.3; 95%CI = 2.3–15.5, respectively).	• Sampling from a single hospital • Sampling from a hospital-based population • Limited sample size	4/10
14. Toorang et al. (2021)	• Higher consumption of sucrose (OR = 2.94; 95%CI = 1.66–5.19), protein (OR = 2.04; 95%CI = 1.17–3.55), saturated fatty acids (OR = 2.21; 95%CI = 1.26–3.87), monosaturated fatty acids (OR = 1.89; 95%CI = 1.09–3.26), and cholesterol (OR = 2.22; 95%CI = 1.28–3.85) were associated with higher risk of stomach cancer. • While higher percent calories from carbohydrates were protective against stomach cancer (OR = 0.57; 95%CI = 0.33–0.98), higher percent calories from proteins were associated with stomach cancer (OR = 3.09; 95%CI = 1.69–5.61).	• Limited sample size • Questionnaire-related biases (e.g., recall bias)	5/10
15. Fararouei et al. (2019)	• Usage of OCPs (OR = 1.77; 95%CI = 1.32–2.38), smoking (OR = 2.48; 95%CI = 1.56–3.96), and exposure to passive smoking (OR = 1.71; 95%CI = 1.28–2.27) were associated with higher likelihood of breast cancer. • Vigorously intense physical activity was protective against breast cancer (OR = 0.68; 95%CI = 0.47–0.98). • Higher consumption of fruits (OR = 1.84; 95%CI = 1.04–1.28), red meat (OR = 1.15; 95%CI = 1.04–1.28), and fish (OR = 1.55; 95%CI = 1.12–2.76) were associated with higher risk of breast cancer. • Higher consumption of pickles was protective against breast cancer (OR = 0.46; 95%CI = 0.31–0.70).	• Sampling from a single hospital • Sampling from a hospital-based population • Questionnaire-related biases (e.g., recall bias)	7/10
16. Hajjar et al. (2022)	• Higher RFS was protective against breast cancer (OR = 0.31)	• NA	6/10
17. Habibi et al. (2019)	• Serum aflatoxin B1 were significantly higher in patients with HCC than patients with chronic viral hepatitis.	• Limited sample size	7/10
18. Ghiasvand et al. (2012)	• Above normal BMI ranges (25.0–29.9) and (≥ 30.0) were associated higher risk of breast cancer (OR = 1.39; 95%CI = 1.02–1.94 & OR = 1.61; 95%CI = 1.18–2.30, respectively).	• Sampling from a single hospital • Sampling from a hospital-based population • Recall bias	8/10
19. Dosemeci et al. (1997)	• The combined effect of alcohol consumption and cigarette smoking was associated with laryngeal cancer (OR = 12.2; 95%CI = 3.1–57.6). • The combined effect of alcohol consumption and cigarette smoking was associated with lung cancer (OR = 14.1; 95%CI = 3.9–61.2).	• Sampling from a single hospital • Sampling from a hospital-based population • Male patients only	5/10
20. Shivappa et al. (2017)	• Higher DII scores were associated with colorectal cancer (OR = 2.13; 95%CI = 1.23–3.72).	• Sampling from a single hospital • Sampling from a hospital-based population • Limited sample size • Recall bias	6/10
21. Simonian et al. (2018)	• BMI (OR = 1.09; 95%CI = 1.03–1.15) and physical inactivity (OR = 36.09; 95%CI = 10.94–119.0) were associated with higher risk of colorectal cancer. • Consumption of NSAIDs were protective against colorectal cancer (OR = 0.34; 95%CI = 0.19–0.62).	• Sampling from a single hospital • Sampling from a hospital-based population • Limited sample size • Recall bias	6/10
22. Rafeemanesh Noet al. (2018)	• Smoking was associated with increased likelihood of breast cancer (OR = 7.14; 95%CI = 2.63–20.0).	• Sampling from a single hospital • Sampling from a hospital-based population • Limited sample size	4/10
23. Ghoreishy et al. (2021)	• Higher dietary phytochemical index was protective against breast cancer (OR = 0.40; 95%CI = 0.26–0.60).	• Questionnaire-related biases (e.g., recall bias)	5/10
24. Kaya et al. (2002)	• Neither anti hepatitis C levels (OR = 1; 95%CI = 0.61–16.44) or hepatitis G RNA (OR = 5.30; 95%CI = 0.61–48.39) were significantly more present patients with non-Hodgkin lymphoma than their control counterparts.	• Limited sample size • Undisclosed controlling techniques	5/10
25. Mazdak et al. (2012)	• Increased consumption of tomato sauce was protective against prostate cancer risk (OR = 2.5; 95%CI = 1.1–2.9). • Smoking, alcohol consumption, intake of garlic, and intake of fat were not associated with prostate cancer risk.	• Sampling from a single hospital • Sampling from a hospital-based population • Limited sample size • Poor controlling of participant characteristics	4/10
26. Rezaeian et al. (2012)	• Hepatitis G genome levels were not significantly different among patients with non-Hodgkin lymphoma compared to controls (OR = 2.31; 95% = 0.28–35.71). • Patients with non-Hodgkin lymphoma had significantly greater levels of hepatitis C genome levels (OR = 1.16; 95%CI = 1.06–1.28).	• Sampling from a single hospital • Sampling from a hospital-based population • Limited sample size	5/10
27. Khodavandi et al. (2011)	• Hepatitis G genome levels were not significantly different among patients with non-Hodgkin lymphoma compared to controls (OR = 2.92; 95% = 0.26–32.25). • Patients with non-Hodgkin lymphoma had significantly greater levels of hepatitis B genome levels (OR = 6.32; 95%CI = 1.30–30.30).	• Unknown sampling frame • Limited sample size	4/10
28. Quadri et al. (2019)	• Consuming “Shammah” was associated with a higher risk of developing oral cancer (pOR = 38.7; 95%CI = 19.5–76.9).	• Limited sample size • Poor quality of evidence	6/11
29. Taha & Eltom (2018)	• Obesity might be implicated within the development of breast cancer.	• Limited sample size • Poor quality of evidence • Qualitative conclusions	5/11
30. Tanner & Cheung (2020)	• Obesity and physical inactivity are probable risk factors for breast cancer development.	• Limited generalizability • Qualitative conclusions	4/11
31. Al-Jaber et al. (2016)	• Tobacco, both cigarette and smokeless, alcohol drinking, and solar radiation exposure are associated with oral cancer.	• Limited sample size • Poor quality of evidence • Qualitative conclusions	5/11
32. Alqahtani et al. (2020)	• HPV infections and smoking were associated with the development of oropharyngeal cancers.	• Questionable statistics	5/11
33. Obeid et al. (2020)	• HPV prevalence among cervical cancer in the MENA region was 81% (95% CI = 70– 90%). • HPV prevalence among samples with abnormal cervical cytology was 54% (95% CI = 40– 67%). • HPV prevalence among the general population at the MENA region was 16% (95% CI = 14– 17%).	• High heterogeneity • Limited representation of MENA countries	9/11
34. Etemadi et al. (2017)	• Current cigarette smoking is associated with higher cancer-related mortality (HR = 1.75; 95%CI = 1.37–2.25). • Current “nass” smoking is associated with higher cancer-related mortality (HR = 1.40; 95%CI = 1.01–1.95). • Combined current cigarette and “nass” are associated with higher cancer-related mortality (HR = 1.67; 95%CI = 1.02–2.75). • Combined former cigarette and “nass” are associated with higher cancer-related mortality (HR = 1.65; 95%CI = 1.13–2.39).	• Incomplete assessment of confounders • Not accounting for smokers quitting through the prospective follow-up period	7/11
35. Charafeddine et al. (2017)	• Tobacco smoking was related to lung, laryngeal, and bladder cancers for both males and females. • Higher BMI was associated with EGCA, endometrial, and renal cancers for females. It was also associated with EGCA, liver, and colon cancers for males. • Low physical activity was associated with gastroesophageal cancer for both males and females. • Alcohol consumption was associated with oropharyngeal and esophageal cancers for both males and females. • Low adherence to Mediterranean diet and H.pylori infection are associated with gastric cancer for both males and females. • Air pollution was associated with lung cancer for both males and females.	• Dependance on other reports • Lack of confidence intervals	4/11
36. Dar et al. (2015)	• Diagnosis of OSCC at a young age was consistent for both regular narghile smokers and occasional narghile smokers.	• Low level of evidence	4/11
37. Wang et al. (2021)	• Higher tertiles of HEI-2015 (OR = 0.22; 95%CI = 0.08–0.60) were protective of lung cancer in current smokers. • Higher tertiles of DASH-Fung (OR = 0.59; 95%CI = 0.38–0.93) were protective of lung cancer. • Higher tertiles of AMED was associated with higher risk of lung cancer among none-smokers (OR = 3.28; 95%CI = 1.50–7.13).	• Limited sample size • Questionnaire-related biases (e.g., recall bias)	7/11
38. Harfouch et al. (2022)	• Occupational status was associated with colorectal cancer.	• Sampling from a single hospital • Sampling from a hospital-based population	6/11
39. Mhawej et al. (2018)	• Tobacco usage and alcohol usage was associated with oropharyngeal squamous cell carcinoma.	• Limited sample size	7/11
40. Abdel-Salam et al. (2020)	• Smokers have a 291% increased risk of death from lung cancer compared to non-smokers (HR = 3.91; 95% HR = 1.66–9.18).	• Sampling from a single hospital • Sampling from a hospital-based population • Limited sample size • Incomplete assessment of confounders	6/11
41. Al-Amad et al. (2014)	• Regular “Narghile” smoking was associated with lower age at diagnosis for oral cancer. • Occasional “Narghile” smoking was associated with lower age at diagnosis for oral cancer.	• Limited sample size • Questionnaire-related biases (e.g., recall bias)	5/8
42. Khodakarami et al. (2012)	• HPV 16 and 18 accounted for 82.2% (95%: 67.9–92.0) of ICC cases.	• Limited sample size	7/8

**Fig. 5:**
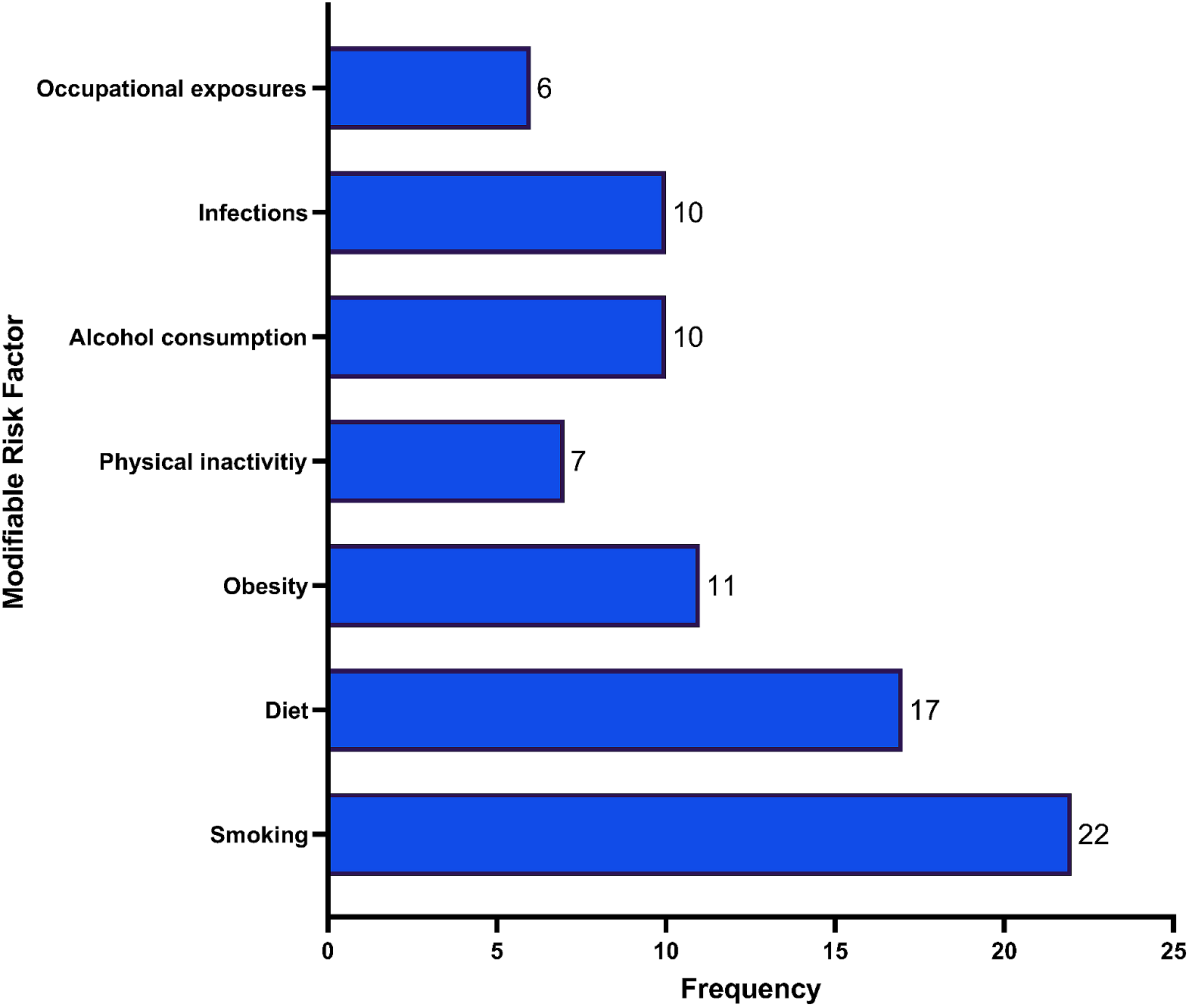
Frequency of studied modifiable risk factors

Most of the studies used a case-control design, with 64.29% being case-control studies, 16.67% being cohort studies, and 14.29% being secondary researches (i.e., narrative reviews, systematic reviews, or meta-analyses) [11, 56–60]. The review found that 22 studies reported tobacco consumption as a predisposing factor for developing cancer, with lung, bladder, squamous cell carcinoma, and colorectal cancer being the most common types of cancer attributable to tobacco consumption in the MENA region (Refer to Fig. 5).

### Smoking and cancer in the middle East

Tobacco consumption was the most investigated risk factor predisposing to cancer across the MENA region. For instance, Abdel-Salam et al. (2020) found tobacco smoking status as the leading predictive factor for lung cancer development [51], while Sasco et al. (2002) reported an increased risk of lung cancer through passive smoking [54]. Nasher et al. (2014) found that the consumption of “Shammah” significantly increased the chances of developing oral squamous cell carcinoma (OSCC) [53]. Additionally, Quadri, Tadakamadla, and John (2019) conducted a systematic review and found a strong link between “Shammah” use and the risk of oral cavity cancer [11]. However, the quality of the studies included in this review was low to moderate.

Bedwani et al. (1997) studied the odds ratio of developing bladder cancer in relation to smoking frequency and found that the risk was higher for those who smoked 20 or more cigarettes per day [43]. Zheng et al. (2012) also reported that smokers of 20 packs of cigarettes per day had a five-fold chance of having urothelial carcinoma compared to non-smokers [44]. The study also concluded that passive smoking through environmental exposure increased the risk of developing urothelial carcinoma but did not affect the squamous cell carcinoma type of bladder cancer.

Some studies explored the combined effect of consuming different types of tobacco. Nasrollahzadeh et al. (2008) showed that cigarette smoking alone had a lower adjusted odds ratio (AOR) than hookah smoking alone, but combining the two reduced the AOR even further [30]. However, Zheng et al. (2012) reported that smoking cigarettes alone had a lower odds ratio (OR) for urothelial carcinoma than in combination with waterpipe smoking [44]. Most of the studies that showed tobacco consumption as a predisposing factor for cancer were country-specific, and no study in the review connected tobacco consumption with cancer in Lebanon, despite its high prevalence of smoking.

### Diet and cancer in the middle East

Research on diet and cancer in the Middle East has shown that specific diets are linked to cancer development, while others are considered protective. A Mediterranean diet, rich in vegetables, fruits, dairy products, fish, seafood, and coffee and tea, has been found to reduce the likelihood of breast cancer in the region. However, a shift from the Mediterranean to a “westernized diet” of fast foods has been reported in earlier literature. There is a need for further research to determine the effect of combining calorie-dense foods with the Mediterranean diet, especially in countries such as Lebanon, where affordability is a concern.

Azzeh et al. (2022) found that consuming three to five portions of animal products weekly, drinking more than three cups per day of tea, three to five servings per week of fish and seafood, more than three cups of coffee per week, five portions of legumes or more every week, and consuming more than three to five servings of fruits and vegetables per week significantly reduced the likelihood of breast cancer [48]. However, the study only involved post-menopausal women, limiting the generalizability of the findings to other populations.

Salarabadi, Bidgoli, and Madani (2015) found that increased use of animal oils and reduced consumption of fish oil, milk, yogurt, white meat, soy, and nuts increased the likelihood of breast cancer, whereas a diet composed of lettuce, cabbage, and carrots reduced the risk [32]. Both studies highlighted the importance of encouraging the consumption of the Mediterranean diet in the Middle East.

Hosseini et al. (2022) used the Food Quality Score (FQS) to assess the nutritional quality of different individual types of food and their link with breast cancer risk [27]. The study found an association between compliance with FQS and breast cancer risk among premenopausal women but not among postmenopausal women. Ghoreishy et al. (2021) employed the dietary phytochemical index (DPI) to determine the impact of diet quality on breast malignancy development [6]. The study found that participants with a high DPI had reduced chances of developing breast cancer compared to those with the lowest DPI.

Wang et al. (2021), Shivappa et al. (2017), and Toorang et al. (2021) investigated the effect of diet on the risk of developing other types of cancer besides breast cancer [34, 35, 42]. Toorang et al. (2021) found that increased consumption of sucrose, proteins, and cholesterol increased the chances of developing stomach cancer, while increasing the number of calories from carbohydrates was protective against stomach cancer development [34]. Wang et al. (2021) linked the Dietary Approach Stop Hypertension (DASH)-Fung score with lung cancer risk, showing an inverse relationship [35]. 

In conclusion, diet affects the development of breast cancer and multiple types of cancer in the Middle East. More studies employing validated diet scores, such as the Mediterranean Diet Score (MDS), Diet Quality Index (DQI), and the Health Eating Index (HEI), are needed to evaluate the effect of diet quality on cancer development in the region.

### Obesity and cancer

The Middle East holds an overrepresented position in the global top obesity ranking. Evidence associating being overweight (BMI > 25) and obese (BMI > 30) with cancer development is weak, with five out of 37 studies linking increased BMI to malignancy [24, 33, 45, 47, 57]. Ghiasvand et al. (2012) found that overweight (BMI > 25–29) or obese (BMI > 30) post-menopausal women over 58 years were at higher risk of developing breast malignancy than those with average weight (BMI 18.5–25) [24]. Elkum et al. (2014) studied the impact of obesity on breast malignancy, including women above 18 years, and found that individuals with a BMI greater than 25 were twice as likely to develop breast malignancy than those with a BMI between 20 and 25, for both premenopausal and post-menopausal women [47]. 

However, the reliability of BMI as a sole parameter for predicting obesity and overweight among women is questionable, with reduced sensitivity and specificity in diagnosing obesity among women [24, 61]. There is a scarcity of studies linking obesity to the risk of developing breast cancer and all types of cancer.

### Physical inactivity and cancer

In this review, few studies linked physical inactivity with the risk of developing cancer in the MENA region. Harfouch et al. (2022) identified higher prevalence of colorectal cancer among inactive professions, [55] while Fararouei et al. (2019) showed that vigorous physical activity lowered the chances of breast malignancy in post-menopausal women [23]. Simonian et al. (2018) recorded similar findings, noting that participants without job-related physical activity have higher chances of developing colorectal malignancy [33]. 

Although Tanner and Cheung (2020) established a relationship between obesity, physical inactivity, and breast malignancy, none of the studies directly linked physical activity to colorectal malignancy [57]. Charafeddine et al. (2017) found that physical activity had less than a 20% protective effect on certain cancers among males, while low physical activity was linked to 21% of gastroesophageal cancer among females in the MENA region [45]. 

Barriers to physical activity in the Middle East include harsh weather conditions, cultural norms, shortage of sports facilities, poor social support, and motivation (Chaabane et al., 2021) [14]. More studies are needed to increase evidence linking physical inactivity and cancer in this region.

### Alcohol consumption and cancer

Although alcohol consumption is relatively low in the MENA region, it remains a common risk factor for cancer development. Studies show increased risks for various cancers among alcohol consumers, such as pancreatic, oropharyngeal, esophageal, liver, and colorectal cancers [45, 46, 49, 62]. Heavy alcohol consumption is more prevalent among men, while women tend to be non-drinkers or occasional drinkers in the region. Alcohol can also increase cancer risk among cigarette smokers, as shown in a study with oropharyngeal squamous cell carcinoma participants [46]. However, some research, such as Nasrollahzadeh et al. (2008), found no connection between alcohol consumption and esophageal squamous cell carcinoma [30]. Nonetheless, the study faced limitations due to participants’ illiteracy, which may have underestimated the significance of alcohol exposure as a potential risk factor.

### Infections and cancer

A few studies in the MENA region have reported a statistically significant relationship between infections and the risk of cancer. For instance, patients with gastric cancer were more likely to have Helicobacter pylori infections [29]. Schistosomiasis was found to increase the likelihood of developing urothelial and squamous cell carcinoma in both men and women [44]. However, no relationship was found between Epstein-Barr virus, human papillomavirus, and oral squamous cell carcinoma [53]. Similarly, there were no significant difference in the presence of hepatitis G or C among non-Hodgkin’s lymphoma patients than controls [50]. On the other hand, two Iranian reports reported conflicting results as they demonstrated that there was no difference in hepatitis G genome among non-Hodgkin lymphoma patients versus controls; however, patients with non-Hodgkin lymphoma had significantly higher concentrations of hepatitis C and B genomes.

Obeid et al. (2008) conducted a meta-analysis which demonstrated that the HPV prevalence within the MENA region was 16% among the general population, with 54% found among patients with abnormal cervical cytology, and 81% among those with cervical cancer [60]. 

### Exposure to environmental carcinogens

The current review found limited literature on environmental carcinogens as a modifiable risk factor for cancer in the MENA region. One study showed that exposure to high levels of heavy metals, such as nickel and chromium, was linked to increased head and neck malignancy [52]. Another study from Eastern Turkey found high amounts of heavy metals like lead, copper, and cadmium in soil, fruits, and vegetables, suggesting that environmental carcinogens might significantly contribute to cancer pathogenesis, although it remains a modifiable risk factor [63]. 

## Discussion

The current scoping review aimed to find the causal relationship between modifiable risk factors and the likelihood of developing cancer in the MENA region. The WHO states that 30–50% of cancers are preventable through healthy lifestyle choices, and others can be cured if detected early. Modifiable risk factors can be grouped into metabolic, behavioral, and environmental. The Global Burden of Disease (2022) highlights that global cancer mortality rates and disability-adjusted life years (DALY) are attributable to behavioral risk factors such as cigarette smoking, risky sexual behavior, and alcohol consumption [64]. 

In the MENA region, smoking, alcohol consumption, high BMI, and exposure to environmental carcinogens are the leading modifiable risk factors causing the most significant burden of cancer and DALYs. The most significant portion of lung malignancy in the MENA region directly correlates with the consumption of tobacco products [11, 65, 66]. The WHO (2015) reported that the Middle East experienced a significant increase in tobacco consumption over the past decade, with Lebanon having the highest per capita cigarette smoking rate globally [67]. In fact, Shisha smoking is a social-cultural practice among people in the Arab World and has been consistently linked to lung, bladder, nasopharyngeal, and esophageal cancer [68]. 

Other reports analyzing data from the Global Burden of Disease datasets of 2019 demonstrated that the incidence and DALYs attributed to various cancers, are on the rise within the MENA region [10, 16, 17]. The most commonly reported risk factors were smoking, high fasting plasma glucose, high BMI, occupational exposure to toxins, and dietary habits. Similar findings were reported in studies examining GLOBOCAN data across the Eastern Mediterranean region or the Gulf Cooperation Council member countries [8, 9]. Across the aforementioned reports, one notion remains consistent; that is, the popularity of smoking and its dominant impact on attributable risk of cancer. The MENA region is currently a rapidly growing market for tobacco consumption [69]. While some nations are trying to implement and cover the WHO’s framework convention on tobacco control and other preventive measures (e.g., quitting clinics, sponsorship bans, taxation among others), the popularity of smoking is increasing multiple folds over the past decade and is even losing its historical ‘taboo’ status among women [9]. Thus, it is only expected for smoking to be the risk factor associated with the highest risk of cancer across the entire region as per the aforementioned published statistics.

However, it should be noted that secondary reports based on either the Global Burden of Disease or GLOBOCAN data might be associated with significant heterogeneity, particularly when it comes to data quality for certain countries. Secondly, most of the published estimates adopt a lax approach when setting assumptions as to simplify their observations (e.g., assuming that risk factors are independent of one another). This issue leads to the inability to assess the joint impact of multiple risk factors or even the confounding effects among them.

To mitigate cancer prevalence in the Middle East, policies should prioritize the reduction of modifiable risk factors. The majority of studies in the current scoping review originated from Iran, indicating a need for more comprehensive research across other countries within the region. Conducting additional studies in diverse nations will contribute to more conclusive findings applicable to the entire MENA region.

Future research should focus on cohort studies, experimental studies using animal models, and more systematic literature reviews. The studies linking predisposing factors to the risk of developing cancer did not have a gender balance as most participants were male. The review mapped many studies showing the association between smoking, high BMI, diet, physical activity, infections, environmental exposure to carcinogens, and the propensity of growing cancer in the Middle East.

The findings justify intensifying efforts to reduce tobacco consumption in the MENA region, such as developing policies that restrict tobacco product consumption. The establishment of physical activity facilities is vital to reduce the number of people at risk of developing obesity, which is a common predisposing factor for breast malignancy. Encouraging the consumption of the Mediterranean diet can also help curb the development of malignancy in this region.

### Limitations

The study findings are subject to some limitations. Firstly, all of the included evidence is mainly comprised of observational studies that are associated with poor-to-moderate methodological quality. Secondly, the sample sizes for such types of associations are too limited to provide accurate associations. Thirdly, there is significant heterogeneity among included studies. While some papers provided associations adjusted for a variety of factors, others fail to account for a number of confounders (e.g., type and duration of smoking). Fourthly, diet-related risk factors were mostly generated through questionnaire-based instruments which introduces recall bias; a bias that is augmented over large periods of time. Fifthly, barely any studies provided sufficient details on their sampling strategies; thus, predisposing them to selection bias. Sixthly, among case-controls, the control subjects were sampled from hospital-based settings which may not be reflective of the general targeted populations. Finally, there is a clear underrepresentation of many Middle Eastern nations which makes generalizing any of the synthesized results or conclusions extremely challenging.

## Conclusion

The quality and quantity of studies reporting on association between modifiable risk factors and cancer within the MENA region are unsatisfactory. Future efforts should supply the need for prospective cohort and experimental studies that attempt to prove the temporal effects of modifiable risk factors on various types of cancer within a MENA cultural and environmental context. Additionally, policy makers should intensify their efforts in promoting and implementing preventive measures against smoking and other prominent modifiable risk factors. Researchers should strive to examine samples that are representative of their original populations across a variety of settings. Also, underrepresented nations within the MENA region should strive to encourage and produce enough scholarly output for the reliable estimation of the impact of modifiable risk factors on cancer.

### Electronic supplementary material

Below is the link to the electronic supplementary material.

**Supplementary Material 1: Supplementary File A.** Detailed search strategy

## Data Availability

The datasets used and/or analyzed during the current study are available from the corresponding author on reasonable request.
