# Supplementary material for: Modifiable risk factors for cancer in the middle East and North Africa: a scoping review
Source: BMC Public Health. 2024 Jan 18;24:223. doi: 10.1186/s12889-024-17787-5 (PMC10797965; doi:10.1186/s12889-024-17787-5)
Supplement: Supplementary file 1 — Supplementary Material 1: Supplementary File A. Detailed search strategy [file 12889_2024_17787_MOESM1_ESM.docx]

**Modifiable Risk Factors for Cancer in the Middle East and North Africa: A scoping Review**

**Supplemental File A: Detailed Database Search Strategy**

***EMBASE***

("Risk factors" OR "Modifiable risk factors" OR "Determinants" OR "Contributing factors") AND

("Cancer" OR "Neoplasms" OR "Malignancy" OR "Tumors") AND

("Adults" OR "Adulthood" OR "Elderly" OR "Youth" OR "Young adults") AND

("Middle East" OR "Eastern Mediterranean" OR "Arab countries" OR "Near East" OR "MENA") AND

("Gulf Council Countries" OR "GCC" OR "Gulf Cooperation Council" OR "Arabian Peninsula") AND

("Arab World Countries" OR "North Africa" OR "Maghreb" OR "Arab nations") AND

("Behavioral risk factors" OR "Environmental risk factors" OR "Lifestyle factors" OR "Health behavior") AND

("Adult"[Mesh] OR "Youth"[Mesh] OR "Elderly"[Mesh] OR "Aged"[Mesh]) AND

("Middle East"[Mesh] OR "Eastern Mediterranean"[Mesh] OR "Arab World"[Mesh]) AND

("Gulf States"[Mesh] OR "Gulf Cooperation Council"[Mesh] OR "Arabian Peninsula"[Mesh]) AND

("Risk*"[Truncation] OR "Modifiable risk factors") AND

("Cancer" OR "Neoplasms" OR "Malignancy" OR "Tumors") AND

("Adults" OR "Youth" OR "Young adults") AND

("Middle East" OR "Gulf*" OR "Arab*") AND

("Risk Factors"[Mesh] OR "Behavioral Risk Factor"[Mesh] OR "Determinants"[Mesh]) AND

("Neoplasms"[Mesh] OR "Malignancy"[Mesh] OR "Tumors"[Mesh]) AND

("Adult"[Mesh] OR "Youth"[Mesh] OR "Elderly"[Mesh]) AND

("Middle East"[Mesh] OR "Gulf States"[Mesh] OR "Arab World"[Mesh]) AND

("1997-01-01"[Date - Publication] : "2022-12-31"[Date - Publication])

***Science Direct***

("Risk factors" OR "Modifiable risk factors" OR "Determinants" OR "Contributing factors" OR "Behavioral risk factors" OR "Environmental risk factors" OR "Lifestyle factors" OR "Health behavior") AND

("Cancer" OR "Neoplasms" OR "Malignancy" OR "Tumors" OR "Oncology") AND

("Adults" OR "Adulthood" OR "Elderly" OR "Youth" OR "Young adults") AND

("Middle East" OR "Eastern Mediterranean" OR "Arab countries" OR "Near East" OR "MENA") AND

("Gulf Council Countries" OR "GCC" OR "Gulf Cooperation Council" OR "Arabian Peninsula") AND

("Arab World Countries" OR "North Africa" OR "Maghreb" OR "Arab nations") AND

("1997-01-01"[Date - Publication] : "2022-12-31"[Date - Publication])

***PubMed***

("Risk factors" OR "Modifiable risk factors" OR "Determinants" OR "Contributing factors") AND

("Cancer" OR "Neoplasms" OR "Malignancy" OR "Tumors") AND

("Adults" OR "Adulthood" OR "Elderly" OR "Youth" OR "Young adults") AND

("Middle East" OR "Eastern Mediterranean" OR "Arab countries" OR "Near East" OR "MENA") AND

("Gulf Council Countries" OR "GCC" OR "Gulf Cooperation Council" OR "Arabian Peninsula") AND

("Arab World Countries" OR "North Africa" OR "Maghreb" OR "Arab nations") AND

("1997-01-01"[Date - Publication] : "2022-08-13"[Date - Publication]) AND

("Risk Factors"[Mesh] OR "Behavioral Risk Factor"[Mesh] OR "Determinants"[Mesh]) AND

("Neoplasms"[Mesh] OR "Malignancy"[Mesh] OR "Tumors"[Mesh]) AND

("Adult"[Mesh] OR "Youth"[Mesh] OR "Elderly"[Mesh] OR "Young Adult"[Mesh]) AND

("Middle East"[Mesh] OR "Eastern Mediterranean"[Mesh] OR "Arab World"[Mesh]) AND

("Gulf States"[Mesh] OR "Gulf Cooperation Council"[Mesh] OR "Arabian Peninsula"[Mesh])

***CINAHL***

S1: ("Risk factors" OR "Modifiable risk factors" OR "Determinants" OR "Contributing factors")

S2: ("Cancer" OR "Neoplasms" OR "Malignancy" OR "Tumors")

S3: ("Adults" OR "Adulthood" OR "Elderly" OR "Youth" OR "Young adults")

S4: ("Middle East" OR "Eastern Mediterranean" OR "Arab countries" OR "Near East" OR "MENA")

S5: ("Gulf Council Countries" OR "GCC" OR "Gulf Cooperation Council" OR "Arabian Peninsula")

S6: ("Arab World Countries" OR "North Africa" OR "Maghreb" OR "Arab nations")

S7: ("1997-01-01"[Date - Publication] : "2022-8-13"[Date - Publication])

S8: (CH "Risk Factors" OR CH "Behavioral Risk Factor" OR CH "Determinants")

S9: (CH "Neoplasms" OR CH "Malignancy" OR CH "Tumors")

S10: (CH "Adult" OR CH "Youth" OR CH "Elderly" OR CH "Young Adult")

S11: (CH "Middle East" OR CH "Eastern Mediterranean" OR CH "Arab World")

S13: S1 AND S2 AND S3 AND S4 AND S5 AND S6 AND S7 AND S8 AND S9 AND S10 AND S11

***Cochrane Library***

(("Risk factors"[Mesh] OR "Risk Factors"[Title/Abstract] OR "Modifiable risk factors"[Mesh] OR "Modifiable Risk Factors"[Title/Abstract] OR "Determinants"[Mesh] OR "Contributing factors"[Mesh] OR "Behavioral risk factors"[Mesh] OR "Environmental risk factors"[Mesh] OR "Lifestyle factors"[Mesh] OR "Health behavior"[Mesh])

AND

("Cancer"[Mesh] OR "Neoplasms"[Mesh] OR "Malignancy"[Mesh] OR "Tumors"[Mesh] OR "Oncology"[Mesh])

AND

("Adult"[Mesh] OR "Adulthood"[Mesh] OR "Elderly"[Mesh] OR "Youth"[Mesh] OR "Young adults"[Mesh])

AND

("Middle East"[Mesh] OR "Eastern Mediterranean"[Mesh] OR "Arab countries"[Mesh] OR "Near East"[Mesh] OR "MENA"[Mesh])

AND

("Gulf Council Countries"[Mesh] OR "GCC"[Mesh] OR "Gulf Cooperation Council"[Mesh] OR "Arabian Peninsula"[Mesh])

AND

("Arab World Countries"[Mesh] OR "North Africa"[Mesh] OR "Maghreb"[Mesh] OR "Arab nations"[Mesh])

AND

("1997/01/01"[Date - Publication] : "2022/12/31"[Date - Publication]))
